# IgG4-Related Retroperitoneal Fibrosis: A Rare Association With Riedel’s Thyroiditis

**DOI:** 10.7759/cureus.13997

**Published:** 2021-03-19

**Authors:** Jonathan C Pacella, Soamsiri Niwattisaiwong, David Newman

**Affiliations:** 1 Pediatrics, University of North Dakota School of Medicine and Health Sciences, Fargo, USA; 2 Endocrinology, Diabetes and Metabolism, Sanford Health, Fargo, USA

**Keywords:** retroperitoneal fibrosis, igg4-related disease, riedel's thyroiditis

## Abstract

Idiopathic retroperitoneal fibrosis is a rare fibro-inflammatory disease that can be associated with other IgG4-related diseases (IgG4-RDs). It is exceedingly uncommon to encounter this condition in a patient with Riedel’s thyroiditis (RT), another disease in the IgG4-RD family. We present the case of a 53-year-old man with a history of RT who presented for severe localized lower abdominal and suprapubic pain due to obstructive uropathy from extensive retroperitoneal fibrosis. The biopsy of the mass demonstrated fibro-inflammatory tissue, and its immunohistochemistry was notable for IgG4-positive plasma cells. This case highlights the challenge associated with the diagnosis and management of this rare manifestation of IgG4-RD. In a patient with a history of any form of IgG4-RDs, providers should be vigilant for any signs or symptoms that suggest the development of fibrosis in other organs.

## Introduction

Retroperitoneal fibrosis is a rare fibro-inflammatory condition that can lead to the entrapment and obstruction of retroperitoneal structures including the ureters and infrarenal vessels such as the aorta and inferior vena cava [1]. It is categorized as primary (idiopathic) or secondary to infections, malignancy, drugs, retroperitoneal hemorrhage, or various other disorders, with the more common idiopathic variant arising in the context of a multifocal fibro-inflammatory disorder recently renamed IgG4-related disease (IgG4-RD) [2]. Imaging modalities such as computed tomography (CT) and magnetic resonance imaging (MRI) are the mainstay of diagnosis [3]. Biopsies are frequently obtained due to concern for malignancy [4]. Recently, management has shifted from a primarily surgical approach to that of immunosuppressive-based therapy using prednisone as first-line treatment, which has demonstrated good long-term patient outcomes [5,6].

Riedel’s thyroiditis (RT) is another rare, destructive fibro-inflammatory condition that is a part of the IgG4-RD spectrum and is the most common thyroid manifestation, with five of the six RT samples demonstrating IgG4-positive immunostaining in a Mayo clinic cohort from 1958 to 2008 [7]. In another case series from the Mayo Clinic, 37 patients with RT were identified during a 64-year period [8]. RT presents as a firm thyroid mass associated with compressive symptoms, hypocalcemia and hypothyroidism, and frequently with multiorgan involvement [9]. Definitive diagnosis is established only with histopathology from an open biopsy and diagnostic criteria have been outlined [10,11]. Most patients with RT do not develop other manifestations of destructive fibroses, with multiple studies demonstrating only one-third eventually having fibrosis in other organs [12,13]. Similarly, RT is very uncommonly diagnosed with an established diagnosis of retroperitoneal fibrosis, with an estimated incidence of less than 1% [13]. In this article, we present the case of a patient with a history of RT who developed IgG4-related retroperitoneal fibrosis with significant obstructive uropathy almost four years after the diagnosis and treatment of RT.

## Case presentation

**Figure 1:**
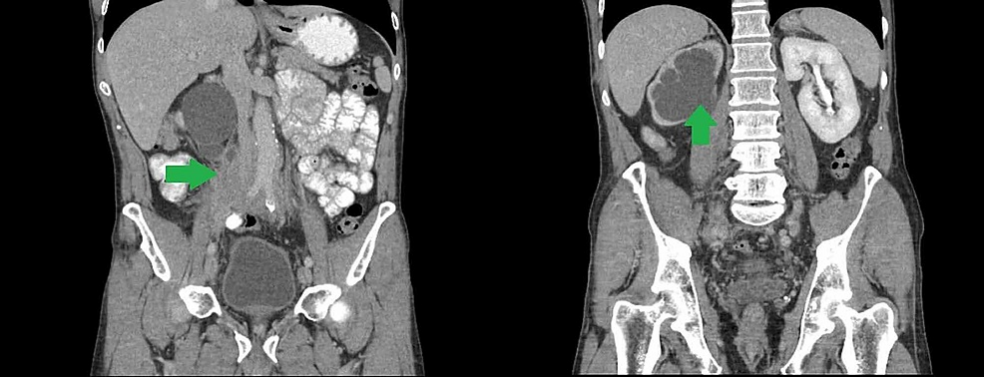
Initial abdominal CT scan. Left: predominantly right-sided retroperitoneal mass encircling the aorta and inferior vena cava. Right: Massive distention of the right pyelocaliceal system and marked loss of renal cortex. CT, computed tomography

**Figure 2:**
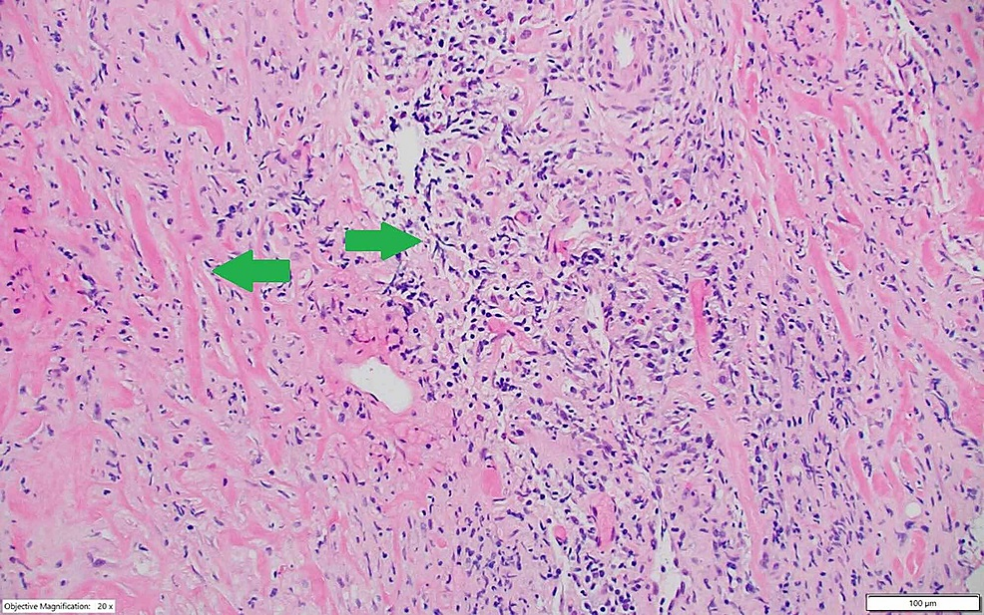
Histopathology. Tissue core from the retroperitoneal mass showing fibrotic tissue (left arrow) with dense lymphocytic infiltrate (right arrow) (H&E, ×100). H&E, hematoxylin and eosin

A 53-year-old male with a history of RT previously treated with isthmectomy for compressive symptom relief presented with a one-week history of severe localized lower abdominal and suprapubic pain. He denied any fever, gastrointestinal symptoms, genitourinary symptoms, or weight loss. He was initially diagnosed with acute prostatitis and was treated with ciprofloxacin without improvement of symptoms, which prompted a second visit to the emergency room. Physical examination demonstrated a flat, soft abdomen with normal bowel sounds and no palpable masses, but with diffuse tenderness across the lower abdomen, especially in the right lower quadrant and suprapubic region. The patient underwent a non-contrast CT scan of the abdomen and pelvis with findings significant for an extensive, predominantly right-sided retroperitoneal mass encircling the aorta, inferior vena cava (Figure 1), and proximal right ureter, producing severe obstructive uropathy of the right kidney with massive distention of the pyelocaliceal system and proximal ureter and marked loss of renal cortex (Figure 2).

**Figure 3:**
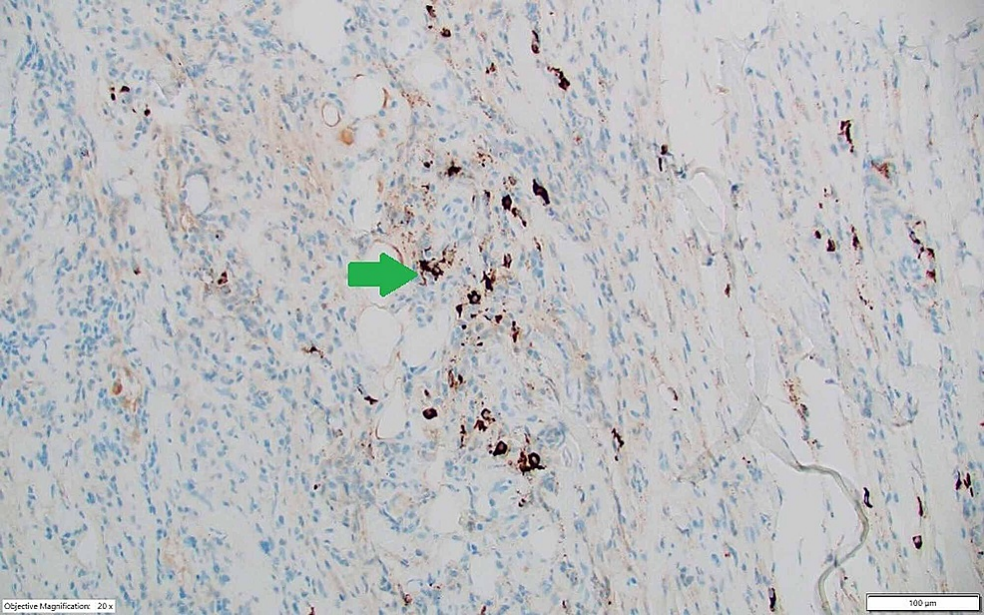
Immunohistochemistry. IgG4 immunohistochemical stain showing 16 IgG4 plasma cells (arrow towards IgG4 staining) per high-power field.

The patient underwent right ureteral stent placement, which partially resolved hydronephrosis and restored kidney function. The CT-guided biopsy of the retroperitoneal mass revealed fibro-inflammatory tissue without specific features (Figure 2). The immunohistochemistry staining was notable for IgG4-positive plasma cells and CD68-positive histiocytes (Figure 3).

The patient was finally diagnosed with IgG4-related systemic fibrosclerosis. Additional lab testing showed normal lactate dehydrogenase, uric acid, and IgG4 levels. He was started on high-dose prednisone at 60 mg daily. Throughout this time, he developed acute renal failure requiring additional stent placement by urology, as well as refractory pain necessitating the use of narcotics.

**Figure 4:**
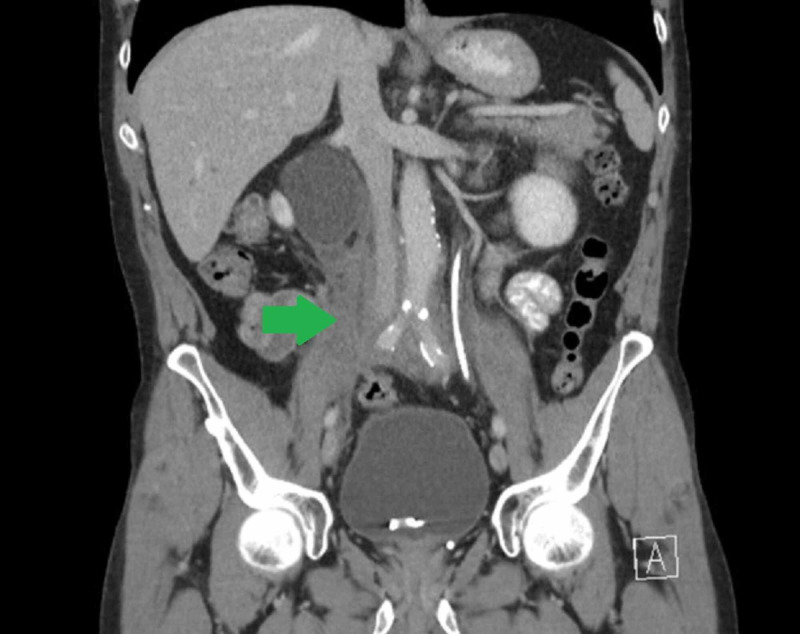
Follow-up abdominal CT scan. Decreased size (7.7 vs 8.1 cm) of the right-sided retroperitoneal mass.

Over the next few months, along with decrease in the size of the retroperitoneal mass on follow-up CT (Figure 4), his analgesic requirements began to decline. His renal function improved and he was able to taper prednisone to a lower dose.

Given the presence of an IgG4-positive plasma cell infiltrate, retroperitoneal fibrosis, and history of RT, the diagnosis of IgG4-related retroperitoneal fibrosis in a patient with RT was made.

## Discussion

IgG4-RD is an immune-mediated fibro-inflammatory condition capable of affecting multiple organs. Its prevalence is unknown, although the recognition of the disease continues to grow [14]. CD4 T cells are likely key players in the disease process, promoting aberrant B cell responses and activating innate immune cells that drive fibrosis [15]. The most common manifestations are organized into four groups, which are pancreato-hepato-biliary disease, retroperitoneal fibrosis and/or aortitis, head-and-neck-limited disease, and classic Mikulicz syndrome (IgG4-related dacryoadenitis and sialadenitis) with systemic involvement [16]. Other symptoms and conditions that can occur include constitutional symptoms, lymphadenopathy, RT, lung and pleural disease, and tubulointerstitial nephritis [16]. The cardinal feature in clinical presentation is single or multiple organ swelling that raises suspicion for malignancy [17]. Retroperitoneal fibrosis can present with poorly localized pain in back, flanks, lower abdomen, or thighs and leg edema or hydronephrosis from ureteral or prostate involvement [17]. At this time, there have been few case studies that have identified retroperitoneal fibrosis in patients with coexistent or resolved RT [18-20].

IgG4-RD requires a broad differential specific to the organ system(s) involved. One of the patient groups that is at high risk of having this disease and requiring further evaluation are those with retroperitoneal fibrosis. Patients with retroperitoneal fibrosis are at a high risk of having IgG4-RD and thus necessitate further evaluation. The diagnostic evaluation of IgG4-RD includes histopathologic, clinical, serologic, and radiologic findings. IgG4 levels are elevated in the majority of patients, although a minority of patients have normal serum levels, as this patient did [17]. Tissue biopsy is a crucial element of the diagnosis and typically demonstrates marked lymphocyte and plasmacyte infiltration, storiform fibrosis, IgG4+ plasma cell infiltration, often accompanied by obliterative phlebitis, and modest tissue eosinophilia [16]. The comprehensive diagnostic criteria from the American College of Rheumatology/European League Against Rheumatism include extensive entry, exclusion, and multiple inclusion criteria that each have a point value [17]. A case of IgG4-RD is diagnosed by the presence of entry criteria, the absence of exclusion criteria, and total inclusion criteria points ≥20 [17]. Using these criteria, given the presence of entry criteria, the absence of exclusion criteria, and a point total of 26, the patient in this case would be diagnosed with IgG4-related retroperitoneal fibrosis.

Primary retroperitoneal fibrosis, whether it is truly idiopathic or associated with IgG4-RD, is treated with immunosuppressive therapy in addition to immediate relief via open surgical, percutaneous, or endoureteral urological interventions if significant obstruction is present. Corticosteroids are considered the first-line therapy [5]. The prognosis of retroperitoneal fibrosis is favorable after initiation of therapy with symptoms, degree of urinary obstruction, and size of the mass typically improving within a few weeks, though full resolution depends on the duration of entrapment [13].

## Conclusions

Retroperitoneal fibrosis is one of the most frequently encountered IgG4-RDs, and RT is the most common thyroid manifestation of these diseases, though Hashimoto’s can exhibit some of the same pathologic features. The two are rarely diagnosed in the same patient. In a patient with the diagnosis of any form of IgG4-RD, clinicians should remain vigilant for any signs or symptoms that suggest the development of fibrosis in other organs. A comprehensive diagnostic criterion is employed to diagnose IgG4-RD, and the patient detailed in this case met the criteria to diagnose the disease. Given his history, the above case likely represents a rare diagnosis of IgG4-related retroperitoneal fibrosis causing obstructive uropathy in a patient with a history of RT whose fibrosis improved with the initiation of corticosteroids.
